# The Characteristics of Syncope-Related Emergency Department Visits: Resource Utilization and Admission Rate Patterns in Emergency Departments

**DOI:** 10.7759/cureus.22039

**Published:** 2022-02-08

**Authors:** Khalid N Almulhim

**Affiliations:** 1 Surgery Department, King Faisal University, Hofuf, SAU

**Keywords:** hospital admission, ed visits, resource utilization, emergency department, syncope

## Abstract

Background and objective

Decision-making about syncope patients presenting to the emergency department (ED) is challenging since physicians must balance the minimal risks of life-threatening conditions with the unessential use of expensive imaging or unnecessary hospitalizations. This study aimed to determine the characteristics of ED visits, resource utilization, and admission rate patterns related to syncope in the United States (US) during the period 2005-2015.

Methods

Data from the National Hospital Ambulatory Medical Care Survey (NHAMCS) on ED visits during the 11-year period from 2005 to 2015 were retrieved. ED visits for syncope were identified and compared against non-syncope ED visits. The demographic and clinical characteristics of patients, as well data on resource allocation and admission trends were captured and described for the syncope and the non-syncope groups.

Results

Syncope accounted for 1.11% of the total ED visits during the study period from 2005 to 2015. The incidence of syncope-related ED visits was higher among elderly females, whites, and non-Hispanics. The trend of admission rates showed a decline from about 30% in 2005-2010 to less than 20% in 2014 and 2015. Advanced imaging (CT or MRI) was ordered for 34% of syncope patients.

Conclusion

The percentage of syncope-related ED visits remained stable during the study period, but the admission rates declined while the use of advanced imaging in syncope-related ED visits remained substantially high despite the advances in research and availability of clinical guidelines. Future research is needed to rationalize healthcare utilization in syncope-related ED visits and precisely identify the high-risk population.

## Introduction

Syncope is defined as a condition characterized by an abrupt, transient, and complete loss of consciousness followed by rapid and spontaneous recovery. Syncope is caused by a period of inadequate cerebral nutrient flow for a short duration (a few seconds), and it most often results from an abrupt drop in systemic blood pressure. Syncope represents a common presentation to emergency departments (EDs) and is estimated to account for 0.6-1.7% of the total ED visits and hospital admission rates of 12-85% [1]. The 30-day mortality rate among syncope patients is about 0.8% [1-6].

The initial evaluation of the symptoms, physical signs, and EKG might be sufficient to reach a diagnosis. However, for many cases, further evaluation might be needed to investigate the cause of syncope and determine the appropriate management plan. Advanced imaging methods such as CT or MRI might be required to exclude central neurological causes of syncope. Although advanced imaging like CT can be useful to detect trauma, bleeding, or stroke in syncope patients, it is not advised to order these diagnostic tests on a routine basis. On the contrary, diagnostic tests for syncope should be guided by clinical information such as patient symptoms and signs, patient history, and physical examination [2,6-8].

Owing to the diagnostic challenges associated with syncope, syncope ED visits might be associated with substantial use of advanced imaging, considerable consultation times, and hospital admissions, which lead to a significant economic burden on healthcare systems [9-11]. At the same time, patients presenting with syncope might have a life-threatening condition and might be at risk of further cardiovascular events and subsequent morbidity and mortality, which pose a challenge for EDs in terms of striking a balance between healthcare utilization and the identification of high-risk individuals for proper management. In their study, Kelly et al. evaluated the frequency and the yield of the pulmonary embolism imaging by CT angiography in syncope patients presenting to the EDs; they found that the CT angiography was frequently performed, although the yield was low [12].

Over the past decade, substantial efforts by medical researchers and professional societies have been undertaken to increase the accuracy of decision-making regarding syncope patients making ED visits. These efforts include the development of new policies and guidelines, developing risk scores to identify at-risk patients, prolonged EKG monitoring for cardiovascular events, and extensive examination and history taking from syncope patients [13-16].

Continuous evaluations of the data of syncope ED visits are needed to examine the temporal trends in resource utilization and determine the impact of the changes on policy and guidelines of the EDs. Several population-based studies have been conducted worldwide and in the United States (US) to evaluate the characteristics and resource utilization of syncope-related ED visits; however, these studies have been limited to a few centers, and have involved short time intervals or old datasets [11,17-19]. In light of this, we conducted this study to describe the demographic and clinical characteristics, admission patterns, and resource utilization pertaining to syncope-related ED visits in the US during the 11-year-period from 2005 to 2015.

## Materials and methods

Study design and setting 

We conducted an analysis of prospectively collected information from a database, the National Hospital Ambulatory Medical Care Survey (NHAMCS), on ED visits during the 11-year period from 2005 to 2015.

Methods of measurement, data collection, and processing

NHAMCS is a prospective multi-center database covering EDs in the US. NHAMCS collects data of the patients presenting to EDs as provided by the physician rather than by the patient; therefore, it provides a nationally representative sample of the US ED visits. Characteristics of each ED visit are entered by the physician via a centralized web-based data management database. Access to the NHAMCS database is provided to participating hospitals through an institution-specific login ID and password. Each hospital is randomly assigned a four-week reporting period. During this period, data for a systematic random sample of visits are recorded by Census interviewers using a computerized Patient Record Form. Data are obtained on patient characteristics such as age, sex, race, and ethnicity, as well as visit characteristics such as patient’s reason for visit, provider’s diagnosis, services ordered or provided, and treatments, including medication therapy. In addition, data about the facility are collected as part of a survey induction interview.

Selection of participants

We selected all adult patients whose reason for ED visits was syncope (syncope group). We compared them against the data of adult patients presenting to the ED for other reasons (non-syncope group).

Data variables

Data variables retrieved from the database and included in this analysis are as follows: (1) patient demographic characteristics: sex, race, ethnicity, age, age group, and payment method; (2) laboratory data: blood urea nitrogen (BUN)/creatine, cardiac enzymes, electrolytes, glucose, basic metabolic panel (BMP), urinalysis, and lactate; (3) imaging/diagnostic data: MRI-head, CT-head, EKG, total number of procedures, pregnancy test, and urine panel; (4) clinical data on visit details, clinical interventions, and chronic diseases; and (5) most common diagnoses related to syncope.

Data analysis

Descriptive statistics were used to describe the characteristics of the two groups. Data were described as mean (±SD) or frequency and as percentages for continuous and categorical variables, respectively. The two groups were compared by the Student's t-test or the Mann-Whitney U test for normally and non-normally distributed continuous variables, respectively. The comparison between the two groups in terms of categorical variables was made using the chi-square test. All analyses were conducted using SPSS Statistics for Windows, version 23 (IBM, Armonk, NY).

## Results

Demographic characteristics of syncope and non-syncope patients

From 2005 to 2015, there were 15,476,451 ED visits related to syncope and 1,395,645,788 visits not related to syncope. Therefore, syncope represented 1.11% of the total ED visits during the study period. In the syncope group, the most common demographic characteristics identified were female gender (58.6%), white race (79.2%), non-Hispanic ethnicity (89.3%), and age group of 18-40 years (32.7%). The demographic characteristics of the syncope and non-syncope patients are shown in Table 1.

**Table 1:** Demographic, laboratory, and imaging characteristics of the syncope and non-syncope ED visits ED: emergency department; BUN: blood urea nitrogen; BMP: basic metabolic panel; MRI: magnetic resonance imaging; CT: computed tomography; EKG: electrocardiogram

Variables		Syncope group (n=15,476,451 visits)	Non-syncope group (n=1,395,645,788 visits)
Sex, %	Female	58.6%	54.8%
	Male	41.4%	45.2%
Race, %	Black	17.9%	23.5%
	White	79.2%	72.9%
	Asian	1.7%	1.9%
	Native American	0.75%	0.43%
	Multiple races	0.26%	0.34%
Ethnicity, %	Hispanic	10.7%	13.9%
	Non-Hispanic	89.3%	86.1%
Age, median		54.4	46.3
Age group, years, %	18-40	32.7%	46.1%
	41-60	26.5%	30.6%
	61-80	26.6%	16.5%
	Over 80	14.2%	6.9%
Payment method	Private insurance	44.4%	37.1%
	Medicare	28.1%	17.7%
	Medicaid	13.9%	27.6%
	Workers comp	0.049%	0.12%
	Self	10.6%	14.2%
	No charge	0.07%	0.14%
	Unknown	3.5%	3.6%
Laboratory data	BUN/creatine	44.4%	19.3%
	Cardiac enzymes	32.6%	9.9%
	Electrolytes	38.3%	15.4%
	Glucose	44.7%	1.8%
	BMP	23.9%	10.5%
	Urinalysis	40.9%	23.4%
	Lactate	0.026%	0.012%
Imaging/diagnostic data	MRI head	0.0081%	0.0027%
	CT head	33.7%	7%
	EKG	72.2%	17.3%
	Total number of procedures	2.7%	2.9%
	Pregnancy test	10.6%	6.1%
	Urine panel	40.92%	23.42%

Clinical characteristics of syncope and non-syncope patients 

Compared to the non-syncope patients, more patients in the syncope group were admitted to the hospital (11.2% vs. 25.5%) and arrived by ambulance (14.9% vs. 49.9%). All recorded chronic diseases such as coronary artery disease (CAD), hypertension (HTN), depression, stroke/cardiovascular disease (CVD), hyperlipidemia, and history of myocardial infarction (MI) were more frequent in the syncope group compared to the non-syncope group (Table 2).

**Table 2:** Clinical characteristics of the syncope and non-syncope ED visits ED: emergency department; AMA: against medical advice; CAD: coronary artery disease; HTN: hypertension; CVD: cardiovascular disease; MI: myocardial infarction; GCS: Glasgow Coma Scale

Variables		Syncope group (n=15,476,451)	Non-syncope group (n=1,395,645,788)
Visit details	Same ED within last 72 hours	0.031%	0.0393%
	Time in same ED in the last 12 months	2.8	2.9
	Left AMA	0.016	0.01
	Alert and oriented x03	74.1	76.6
	Admitted	25.5%	11.2%
	Length of visit in ED (minutes)	294.6	294.6
	Arrival by ambulance	49.4	14.9
	Length of stay in hospital if admitted (minutes)	1119.9	1440.17
	The average number of diagnoses in the ED	6.6	4.5
	Transfer to another hospital	2.9	1.4
	Return/transfer to a nursing home	0.023	0.28
	Wait time to see the physician (minutes)	41.6	45.7
	Number of medications given in the ED	2.7	2.6
	Number of medications given at discharge	0.68	1.1
Chronic diseases	Chronic conditions (mean)	2.1	1.9
	CAD	11.7%	5.4
	HTN	36.1%	22.2
	Depression	9.1%	8.8
	Stroke/CVD	6.1%	2.6
	Hyperlipidemia	13.6%	7.4
	History of MI	3.6%	2.1
Clinical details	IV fluids given	54.3%	25.4%
	Thrombolytic therapy given	0.11%	0.17%
	Initial pulse	88.1	100.6
	Initial pulse (systolic)	133.5	153.5
	Initial pulse (diastolic)	78.5	100.2
	Mean initial GCS	14.5	14.4
	Mean initial pain scale	5.5	6.1

Timing-related characteristics of syncope and non-syncope ED visits

More than 80% of the syncope patients were seen within 60 minutes of their visit to the ED compared to 50% in the non-syncope group. Syncope-related ED visits were slightly higher on Tuesdays and in February (Table 3).

**Table 3:** Timing-related characteristics of the syncope and non-syncope ED visits ED: emergency department

Variables		Syncope group	Non-syncope group
Immediacy with which the patient was seen	Immediately	9.7%	4.6%
	1-14 minutes	24.9%	10.9%
	15-60 minutes	47.9%	36.8%
	>1 hour	10.6%	21.3%
	>2 hours	3.9%	10.6%
	No triage	2.1%	16.6%
Day of the week of the visit	Monday	14.9%	15.6%
	Tuesday	15.4%	14.5%
	Wednesday	15.1%	14.2%
	Thursday	14.4%	13.8%
	Friday	14.9%	13.7%
	Saturday	12.7%	14.1%
	Sunday	12.6%	14.3%
Month of the visit	January	7.9%	8.4%
	February	9.2%	8.1%
	March	8.8%	9.1%
	April	7.9%	8.9%
	May	7.9%	8.8%
	June	8.5%	8.2%
	July	8.6%	8.3%
	August	8.8%	8.6%
	September	8.2	8.3%
	October	8.7	8.4%
	November	8.3	7.9%
	December	7.2	7.2%

Admission rates of syncope patients

**Figure 1:**
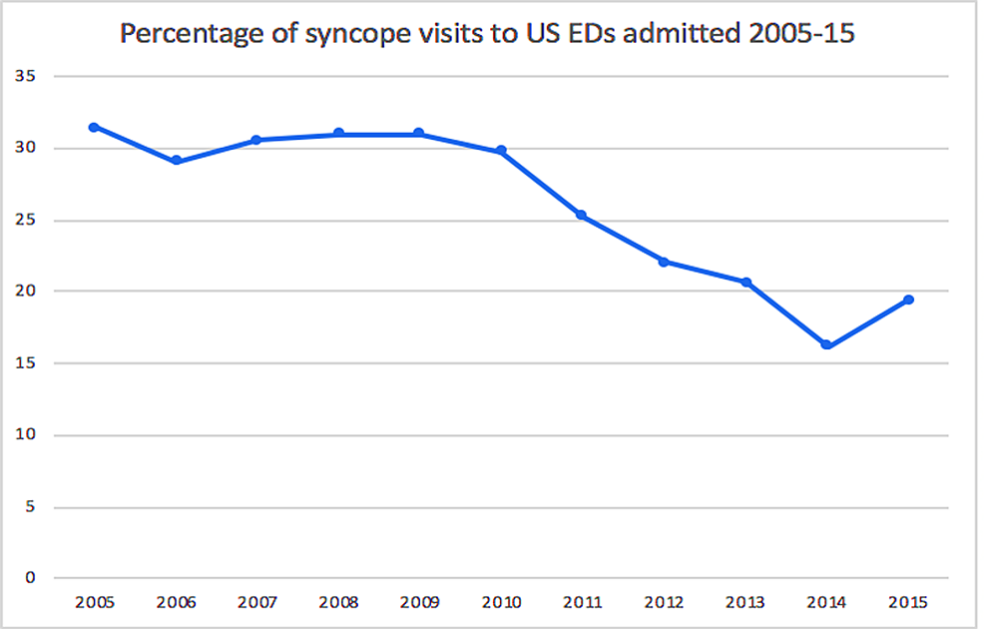
Percentage of syncope admissions from among the total syncope ED visits during the study period (2005-2015) ED: emergency department

The trend of admission rates showed a decline from about 30% in 2005-2010 to less than 20% in 2014 and 2015 (Figure 1).

Common diagnoses related to syncope

The most common diagnoses related to syncope were fainting (40.78%), vertigo (13.39%), general weakness (4.18%), chest pain (4.08%), convulsions (2.41%), headache (2.16%), nausea (1.45%), abdominal pain (1.28%), shortness of breath (1.13%), and back pain (0.52%), as shown in Table 4.

**Table 4:** Most common diagnoses related to syncope in US ED visits ED: emergency department

Diagnosis	Percentage
Fainting	40.78%
Vertigo	13.39%
General weakness	4.18%
Chest pain	4.08%
Convulsions	2.41%
Headache	2.16%
Nausea	1.45%
Abdominal pain	1.28%
Shortness of breath	1.13%
Back pain	0.52%

EKG and head CT in syncope and non-syncope groups

Compared to the non-syncope group, the percentage of EKG taken in the syncope group was six-fold higher in the age group of 18-40 years, three-fold higher in the age group of 41-60 years, and two-fold higher in the +60-year age group (Table 5).

**Table 5:** Frequency of EKG and head CT in the scope and non-syncope groups EKG: electrocardiogram; CT: computed tomography

Age group, years	Syncope group, %		Non-syncope group, %	
	EKG	Head CT	EKG	Head CT
18-40	60.9	26.5	10.0	5.3
41-60	75.3	34.2	23.8	7.8
61-80	84.2	41.9	40.1	12.2
Over 80	86.8	40.2	48.1	19.2

## Discussion

This study aimed to describe the nationwide characteristics of syncope-related ED visits, resource utilization, and admission rate patterns in the US during the period from 2005 to 2015. Evaluating the characteristics, admission rates, and resource utilization related to syncope-related ED visits is important to guide further improvements in ED practice and to help decision-makers rationalize the healthcare costs.

The percentages of the syncope-related ED visits out of the total ED visits in the period 2005-2015 remained stable at about 1.11%. This finding is consistent with the previous analysis by Probst et al. [19] for the 10-year period from 2001 to 2010. However, it is higher than the 0.77% rate reported based on the same database for the period 1992-2000 [20]. Our study showed that a high proportion of syncope-related ED visits involved elderly females, whites, and non-Hispanics, which is similar to the findings of Sun et al. [20] for the 1992-2000 period. These demographics are also consistent with a recent population-based study in Canada that showed a similar higher incidence of syncope in women. However, women were less likely to be admitted and had lower mortality rates compared to men [21].

Our study showed that hospital admission rates related to syncope ED visits decreased during the study period from about 30% in the five-year span of 2005-2010 to less than 20% in 2014 and 2015, which reflects an improvement in the identification of cases that require hospitalization. The overall admission rates of syncope visits for the period 2005-2015 was 25.5% compared to 11.2% for non-syncope visits. Probst et al. [19] have shown that hospitalization rates in 2001-2010 ranged from 27 to 35% and showed no significant downward trend. In another study by Chou et al. [17], hospitalization rates in 2002-2007 remained stable at 37% but declined to 25.7% by 2015. Similar rates were reported by Anderson et al. who analyzed the data from the National Emergency Department Sample from 2006 to 2014 and the State Inpatient Databases and Emergency Department Databases from 2009 and 2013 [18].

In our study, MRI and CT of the head were performed for 0.0081% and 33.7% of the syncope visits respectively, which is very high. For the period 2002-2007, Chou et al. [17] reported an increase in advanced imaging rates from 27.2% to 42.1%, while Probst et al. [19] reported an increase in advanced imaging rates from 21% to 45% for the period 2001-2010. This decline was explained by the increasing recognition of the high healthcare costs associated with advanced imaging use as well as the adverse risks of exposure to radiation [17,22,23]. These rates reflect the need for further improvements with the aid of novel strategies, additional policy changes, and augmentation of society guidelines. Chou et al. [17] investigated whether the trends in admission and imaging rates were related to the introduction of the American College of Emergency Physicians (ACEP) clinical policy in 2007; they found that these trends were similar for the syncope and non-syncope visits, which indicated that these trends were not influenced by ACEP policy but rather reflect a broader practice shifts.

In order to rationalize healthcare costs and ensure better resource utilization, there is a need to identify patients with potentially serious outcomes and safely discharge those who do not require hospitalization. The first step in achieving this is to identify the high-risk population by studying the risk factors of major cardiovascular events and poor clinical outcomes within 30 days of the ED visits. Several studies have described these risk factors, and furthermore, clinical risk scores have been developed to facilitate decision-making in the ED.

In a prospective study in Stanford, mortality rates were low among the 1,418 consecutive patients who visited the ED due to syncope (1.4% at 30 days, 4.3% at six months, and 7.6% at one year) and were predictable by the risk factors (sensitivity 89%) [24]. Gabayan et al. [25] studied the patterns and predictors of short-term cardiac outcomes in ED patients with syncope by using data from an integrated health system of 11 Southern California EDs. During the period 2002-2005, 35,330 syncope-related ED visits were studied for cardiac outcome and risk factors. The rate of seven-day cardiac outcomes was low (3%). Risk factors for cardiac outcomes within seven days of the ED visit were as follows: age ≥60 years, male gender, congestive heart failure, ischemic heart disease, cardiac arrhythmia, and valvular heart disease.

To overcome the challenge of decision-making regarding syncope patients, the Canadian Syncope Risk Score was developed and has been validated as a tool to predict the 30-day serious outcomes not evident during the initial ED evaluation [14]. The risk stratification of old patients by the FAINT score was also proposed and validated [26]. Emergency physicians in San Francisco have developed the “San Francisco Syncope Rule” to recognize patients with potentially serious outcomes; in a prospective study covering the period 2002-2004, this rule showed a 98% sensitivity in identifying patients with serious outcomes [27]. Another solution was proposed by Probst et al., who introduced the approach of shared decision-making for patients with unexplained syncope, and they found that this approach was feasible and increased patient knowledge and involvement; however, their study was not sufficiently powered to evaluate differences from the usual care in terms of the clinical outcomes at 30 days [16]. 

In a prospective study by Morag et al., patients with prior syncopal episodes were followed up after their ED visit [28]. Two-thirds of them were hospitalized. After one month, none of them developed a life-threatening condition, required significant therapeutic intervention, or died; however, recurrent syncope occurred in 2.2% of the patients. These results highlight the importance of negative-structured ED evaluation to identify patients who can be safely discharged to reduce the associated healthcare costs [28]. It was found that adherence to the syncope guidelines helped to improve decision-making, aided in terms of deciding the appropriate disposition of syncope patients, and reduced resource utilization and healthcare expenses [15]. Moreover, prolonged EKG monitoring (>12 hours) of syncope patients was proposed to have high accuracy in identifying the major cardiovascular events seven and 30 days after the ED visit [13].

Our study has several strong points: (1) we used the largest database covering EDs in the United States, (2) we covered a long period of 11 years (2005-2015), and (3) we described the characteristics and resource utilization related to syncope visits, as well as non-syncope visits as a reference for the trends and changes over time. Nonetheless, our study is limited by the lack of data on the clinical outcomes of syncope-related ED visits, and hence we could not describe the frequency of cardiovascular events or mortality after the ED visit. 

Future research is needed to (1) properly identify syncope patients who have life-threatening conditions or are at risk of major cardiovascular events, and (2) develop risk scores and recommendations to reduce health resource utilization and expenditure in the EDs.

## Conclusions

During our study period of 11 years from 2005 to 2015, the percentage of syncope-related ED visits remained stable. Elderly females, whites, and non-Hispanics account for the highest number of syncope-related ED visits in the US. The use of advanced imaging techniques in syncope-related ED visits remained substantially high despite the advances in research and clinical guidelines. Future research is needed to rationalize healthcare utilization in syncope-related ED visits and properly identify the high-risk population.
